# Clinical and Sociodemographic Aspects of Inflammatory Bowel Disease Patients

**DOI:** 10.14740/gr649w

**Published:** 2015-07-22

**Authors:** Leda Maria Delmondes, Marcelo Oliveira Nunes, Arthur Rangel Azevedo, Murilo Matos de Santana Oliveira, Lorena Eugenia Rosa Coelho, Juvenal da Rocha Torres-Neto

**Affiliations:** a Department of Medicine, Tiradentes University, Aracaju 49032-490, Brazil; b Coloproctology Department, Sergipe Federal University, Sao Cristovao 49100-000, Brazil

**Keywords:** Inflammatory bowel diseases, Ulcerative colitis, Crohn’s disease, Epidemiology

## Abstract

**Background:**

In Brazil, there are few epidemiological studies available about the demographic and clinical aspects of inflammatory bowel disease (IBD). The aim of this study was to identify epidemiological and clinical characteristics of patients with IBD treated at the University Hospital (HU) of the Sergipe Federal University (UFS).

**Methods:**

A cross-sectional descriptive study was conducted in HU/UFS from October 2011 to January 2014. The sample consisted of 87 patients with IBD, who registered in the coloproctology clinic. We applied a questionnaire with sociodemographic and clinical variables.

**Results:**

Of the 87 patients, 40 (46%) had Crohn’s disease (CD) and 47 (54%) had ulcerative colitis (UC). Women had a higher prevalence of IBD. Data obtained were significant (P < 0.05) in the variables: age, origin and level of education. CD patients were younger (< 25 years old), had higher prevalence of smoking habits and were associated with urban origin, conjunctivitis, palpable mass, appendectomy and intestinal complications. UC was more prevalent in older individuals (> 25 years old), with rural origin, bloody diarrhea and rectal bleeding. Location and initial behavior of CD were ileum-colic (L3), inflammatory behavior and penetrating form of the disease. There is higher prevalence of proctitis and mild and severe forms of the UC among women. Osteoarticular and systemic manifestations predominated in both diseases.

**Conclusions:**

IBD affected more women than men. The age, origin and level of education can interfere with early diagnosis. Demographic and clinical aspects were similar to the literature. Data differ in the time interval between the onset of symptoms and diagnosis, smoking habit, appendectomy and severity of UC for age and gender.

## Introduction

Inflammatory bowel disease (IBD) is a group of inflammatory diseases that include Crohn’s disease (CD) and ulcerative colitis (UC) and can affect all people. CD preferably affects young people, while UC is bimodal. They affect people from different socioeconomic levels, age, gender, nationality and origin. Although the risk factors are not yet clear, some may be part of the natural history of these diseases [1-3], such as age and gender, race and ethnicity, genetic susceptibility, family history, smoking habits, appendectomy, eating habits, psychosocial factors, obesity and infections, contraceptives and hormone replacement therapy, non-steroidal anti-inflammatory drugs and others.

IBD usually develops with frequent relapses and severe clinical manifestations. The incidence and prevalence of IBD in different geographical regions have been different [4-7]. Prevalence rates may be increasing due to earlier diagnosis and longer duration of disease. Even with recent advances in genetics, its pathogenesis is not yet fully understood [7-11]. Complex interactions between genetic and environmental factors, immunoregulation of the mucosa and intestinal microbiota account for the etiology of these diseases. Evidence shows that personality or psycho-emotional disorders contribute to either triggering the disease or altering the course of its evolution [12-15].

IBD has low mortality rates, high morbidity, and unpredictable clinical complications that may compromise the patients regarding their social and personal performance. The symptoms of both diseases (CD and UC) include most common intestinal manifestations (IMs), such as abdominal pain, chronic diarrhea with or without rectal blood and mucus in feces [1, 6]. CD may affect any part of the digestive tract attacking the entire intestinal wall, but has a predilection for ileal or ileocecal region. Perianal abscess and fistula suggests CD [16, 17]. UC is a cyclical disease, with phases of exacerbation and remission, with a variable degree of intensity and continuously affects the lining of the rectum and colon. Extra-intestinal manifestations (EIMs) may precede the symptoms of the disease [18-20].

In Brazil, few epidemiological studies available show the incidence and prevalence of IBD [4, 21-23]. Due to its chronic unstable characteristics and unknown etiology, identifying the epidemiological and clinical characteristics of this population can promote diagnosis and earlier clinical intervention.

## Materials and Methods

This is a cross-sectional descriptive study conducted at the University Hospital (HU) of the Federal University of Sergipe (UFS) from October 2011 to January 2014 with patients with IBD, who attended to the Clinic of Coloproctology in HU/UFS. Eighty-seven patients out of 89 diagnosed with IBD answered the questionnaire. Both of those who did not respond it had UC. The sample consisted of 87 patients (40 CD and 47 UC). Inclusion criteria were diagnosis of IBD from the combination of clinical, radiological, endoscopic, and histopathological evaluation (mucosal biopsies or surgical resection specimens). Exclusion criteria were colitis from other causes; cancer; HIV/AIDS; bedridden; pregnant women or nursing mothers; treatment of complications of IBD; multiple organ failure; addicts. We used a questionnaire prepared for the purpose of this study.

To identify the sociodemographic profile, the following variables were considered: age, gender, origin (residence location), skin color, number of residents in the same household, education level, household income, employment status and smoking habits. The variables for clinical profile were: onset clinical manifestations and when they started; time between symptoms and diagnosis; family history of IBD; age when symptoms started; initial behavior of CD (inflammatory, stenotic or penetrating); location and extent of CD according to the Montreal classification; complications of perianal CD (abscess, fistula, and fissure); forms of the UC (light/transient, moderate, severe, and other); location and extent of UC (distal, left colitis, pancolitis, and retrograde ileitis); severity of UC correlated to gender and age; IMs; EIMs; and other manifestations of CD and UC.

For data analysis, we used the IBM SPSS Statistics software for Windows version 17. Quantitative variables were expressed as mean ± SD when normally distributed. Simple frequencies and percentages were organized in tables. To evaluate dependence between diseases, the Pearson c^2^ test was applied. The significance level was 5%.

The Ethics Committee on Human Research of the Federal University of Sergipe - CEP/UFS, approves this study (CAAE: 0327.0107.000-11). All individuals signed a consent form.

## Results

Eighty-seven patients were registered with IBD. Forty of them (46%) were diagnosed with CD and 47 (54%) with UC. Some patients did not respond to all of the variables, thus, the percentages were not calculated to the total number of patients in each subgroup in the absence of values. The demographic and socioeconomic characteristics of 87 patients with IBD (Table 1) were significant in the variables: age (P < 0.01), origin (P < 0.03) and educational level (P < 0.01). Age ranged from 16 to 79 years old. The average time length of disease was 10 years. The male/female ratio was 1:1.86 for CD and 1:1.35 for UC. As for educational background, there was a prevalence of high school level education in 23 (58%) with CD and elementary school level in 24 (53%) suffering from UC. The brown color was reported by 50% of respondents. Over 50% of the patients with IBD had some work activity, with family income between 1 and 10 living wages. Regarding smoking habits, 68 (80%) patients were former smokers. These statistical data do not show any significance. There was loss of information, which hindered the possibility of crossing some data.

**Table 1:** Demographic and Socioeconomic Characteristics of IBD Patients

	Diagnosis			
	CD	UC	Total	χ^2^ (P value)
Age				
16 - 30 years old	16 (40)	2 (4)	18 (21)	19.80 (0.01)
31 - 45 years old	14 (35)	21 (46)	35 (40)	
46 - 60 years old	8 (20)	12 (25)	20 (23)	
> 60 years old	2 (5)	12 (25)	14 (16)	
Gender				
Female	26 (65)	27 (57)	53 (61)	0.52 (0.47)
Male	14 (35)	20 (43)	34 (39)	
Origin				
Urban	37 (95)	35 (78)	72 (86)	4.99 (0.03)
Rural	2 (5)	10 (22)	12 (14)	
Skin color				
White	13 (35)	14 (33)	27 (34)	0.44 (0.80)
Black	7 (19)	6 (14)	13 (16)	
Mixed race	17 (46)	22 (53)	39 (50)	
Number of residents in the same household				
One	0 (0)	2 (4)	2 (2)	2.09 (0.55)
Two	9 (25)	8 (18)	17 (21)	
Three	10 (28)	13 (30)	23 (29)	
More than three	17 (47)	21 (48)	38 (48)	
Level of education				
Illiterate	0 (0)	3 (7)	3 (3)	11.07 (0.01)
Middle school	13 (32)	24 (53)	37 (44)	
High School	23 (58)	11 (24)	34 (40)	
College	4 (10)	7 (16)	11 (13)	
Household income*				
Less than 1	3 (8)	4 (9)	7 (8)	5.98 (0.11)
1 - 10	35 (88)	29 (68)	64 (78)	
11 - 15	1 (2)	6 (14)	7 (8)	
More than 15	1 (2)	4 (9)	5 (6)	
Employment				
Employed	17 (43)	23 (50)	40 (47)	7.10 (0.13)
Unemployed	12 (31)	9 (20)	21 (25)	
Household professional	3 (8)	3 (6)	6 (7)	
Student	3 (8)	0 (0)	3 (3)	
Retired	4 (10)	11 (24)	15 (18)	
Smoking habit				
Yes	3 (8)	2 (4)	5 (6)	2.70 (0.26)
Former smoker	33 (84)	35 (76)	68 (80)	
No	3 (8)	9 (20)	12 (14)	

*Household income is measured in Brazilian Minimum Wege (R$788.00/month).

Regarding the clinical manifestations of patients with IBD (Table 2), the age at which the symptoms started was significant (P < 0.01) and the report of appendectomy was unique to CD (P < 0.02). Data revealed no significance as to the onset of clinical manifestations and the time between the onset of symptoms and diagnosis. Sixty-three patients out of 81 respondents reported no first-degree relatives of patients with IBD.

**Table 2:** Clinical Characteristics of IBD Patients

	Diagnosis			
	CD	UC	Total	χ^2^ (P score)
Age when symptoms started				
0 - 15 years old	11 (28)	5 (12)	16 (20)	13.67 (0.01)
15 - 25 years old	10 (25)	2 (5)	12 (15)	
25 - 35 years old	9 (23)	13 (32)	22 (28)	
35 - 45 years old	4 (10)	6 (14)	10 (12)	
> 45 years old	5 (13)	15 (36)	20 (25)	
Time since manifestations started				
0 - 5 years	17 (43)	17 (40)	34 (41)	5.02 (0.29)
5 - 10 years	10 (25)	15 (36)	25 (30)	
10 - 15 years	7 (18)	8 (19)	15 (18)	
15 - 20 years	4 (10)	0 (0)	4 (5)	
> 20 years	2 (5)	2 (5)	4 (5)	
Time between start of symptoms and diagnosis				
0 - 5 months	12 (34)	13 (35)	25 (35)	2.19 (0.82)
5 - 10 months	6 (17)	5 (13)	11 (15)	
10 - 15 months	8 (23)	9 (24)	17 (24)	
15 - 20 months	0 (0)	1 (3)	1 (1)	
20 or more months	9 (26)	8 (22)	17 (24)	
Could not inform	0 (0)	1 (3)	1 (1)	
Family history				
No	28 (72)	35 (83)	63 (78)	4.97 (0.29)
Parents	5 (13)	3 (7)	8 (10)	
Siblings	4 (10)	1 (2)	5 (6)	
Uncles/cousings/nephews	1 (3)	3 (7)	4 (5)	
Grandparents	1 (3)	0 (0)	1 (1)	
Appendectomy history				
Yes	5 (14)	0 (0)	5 (7)	5.95 (0.02)
No	31 (86)	40 (100)	71 (93)	

**Figure 1:**
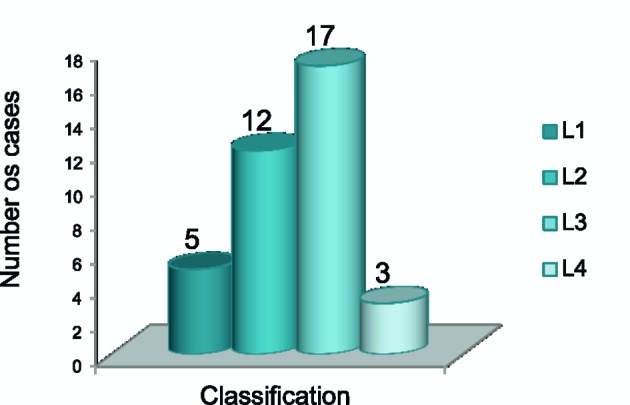
Location (L) of UC according to Montreal classification.

CD patients were grouped according to the Montreal classification (Fig. 1). Among the 40 patients with CD, only 37 had reported the location and extent of disease. Therefore, 17 (47%) were located in the ileocolonic (L3), 12 (32%) in the colon (L2), five (13%) in the ileum (L1) and three (8%) in the upper gastrointestinal tract (L4). Three patients presented manifestation of CD in the upper gastrointestinal tract (duodenum and esophagus).

The clinical characteristics of patients with CD (Table 3) included the type of initial commitment (A), the form of infiltration (B), IMs, EIMs and other events. Abscess (30%) and perianal fistula (30%) were the most prevalent forms of the perforating disease. The patients had more than one MI; the most common were: intestinal colic (62%), abdominal tenderness (60%), watery diarrhea (59%) and mucus in the stool (54%). The presence of palpable mass was significant (P < 0.04). In EIMs osteoarticular (35% arthralgia and arthritis 30%) predominated. The ophthalmological findings were significant (P < 0.04) between CD and UC and only present in patients suffering from CD. Only patients with AD had dermatological and vascular manifestation. One patient had all thromboembolic events: venous, arterial and in the central nervous system. Three patients were affected by hepatic manifestations (cholestasis, sclerosing cholangitis, and hepatitis).

**Table 3:** Clinical Characteristics of CD Patients

CD initial behavior		Superior GI tract manifestations	
Inflammatory (B1)	20 (64)	Duodenal	1 (%)
Stenotic (B2)	4 (13)	Esophagic	2 (%)
Penetrating (B3)	7 (23)	Dermathologic manifestations	
Penetrating forms		Pyodermitis	3 (7)
Abscess	10 (67)	Pyoderma Gangrenosum	3 (7)
Perianal fistula	10 (67)	Erythema nodosum	1 (2)
Rectovaginal fistula	4 (27)	Ophthalmologic manifestations	
Enterocutaneous fistula	2 (13)	Conjuntivitis	4 (10)
Entero-enteral fistula	3 (20)	Uveitis	2 (5)
Enterovesical fistula	1 (7)	Episcleritis	2 (5)
Enteroscrotal fistula	0 (0)	Vascular manifestations	
Intestinal manifestations		Venous thromboembolism in upper limb	1 (2)
Intestinal colics	25 (62)	Venous thromboembolism in lower limb	1 (2)
Abdominal pain	24 (60)	Arterial thromboembolism in upper limb	1 (2)
Tenesmus	17 (42)	Arterial thromboembolism in lower limb	1 (2)
Pasty diarrhea	14 (36)	Tromboembolism in CNS	1 (2)
Liquid diarrhea	23 (59)	Hepatic manifestations	
Bloody diarrhea	17 (44)	Colestasis	1 (2)
Exclusive rectal bleeding	9 (23)	Sclerosing colangitis	1 (2)
Mucus in feces	21 (52)	Hepatitis	1 (2)
Palpable mass	9 (23)	Other manifestations	
Intestinal perforation	3 (9)	Weakness	24 (62)
Intestinal obstruction	1 (3)	Weigh loss (> 10% body weigh)	26 (67)
Osteoarthicular manifestations		Adinamy	23 (59)
Joint pain	14 (35)	Paleness	22 (56)
Arthritis	12 (30)	Subnutrition	17 (46)
Sacroileitis	2 (5)	Vomiting	16 (41)
Ankylosing spondylitis	1 (2)	Pirosis	12 (31)
		Fever	9 (23)
		Dysphagia	3 (8)
		Epigastric pain	14 (36)

Of UC patients, 21 (57%) patients were female and 16 (43%) were male. The clinical features of UC are detailed in Table 4. There was a predominance of mild form (54%) as the initial phase of the disease. Thirty patients (71%) showed the distal location and extension (E1). For IMs, 71% of patients with UC had bloody diarrhea, 71% had mucus in the stool, 67% watery diarrhea, 62% intestinal colic, 62% tenesmus and 51% pasty diarrhea. Exclusive rectal bleeding was prevalent in patients with UC (P < 0.05).

**Table 4:** Clinical Characteristics of UC patients

Clinical presentation of UC		Extra-intestinal manifestations	
Mild/transitional	20 (54)	Osteoarthicular manifestations	
Moderate	11 (30)	Arthritis	9 (21)
Severe	6 (16)	Joint pain	8 (19)
Extension of UC		Sacroileitis	2 (5)
Proctitis (distal) (E1)	30 (71)	Ankylosing spondylitis	1 (2)
Left colits (E2)	6 (14)	Hepatic manifestations	
Pancolitis (E3)	5 (12)	Colestasis	2 (5)
Retrograde iletits	1 (2)	Sclerosing colangitis	2 (5)
Intestinal manifestations		Hepatitis	2 (5)
Intestinal colics	26 (62)	Other manifestations	
Abdominal pain	18 (43)	Weakness	20 (49)
Tenesmus	26 (62)	Weigh loss(> 10% body weight)	24 (57)
Pasty diarrhea	21 (51)	Adinamy	18 (43)
Liquid diarrhea	29 (67)	Paleness	21 (51)
Bloody diarrhea	30 (71)	Subnutrition	14 (37)
Exclusive rectal bleeding	19 (45)	Vomiting	10 (24)
Mucus in feces	30 (71)	Nausea	17 (40)
Palpable mass	1 (2)	Pirosis	14 (33)
		Fever	5 (12)
		Dysphagia	7 (17)
		Epigastric pain	18 (43)

Forty-two patients had EIMs: 12 had osteoarticular manifestations and six patients had hepatic manifestations. Fifteen patients had at least one osteoarticular or hepatic manifestation. Among other manifestations of the disease, weight loss (57%) and pale skin (51%) were the most frequent.

**Figure 2:**
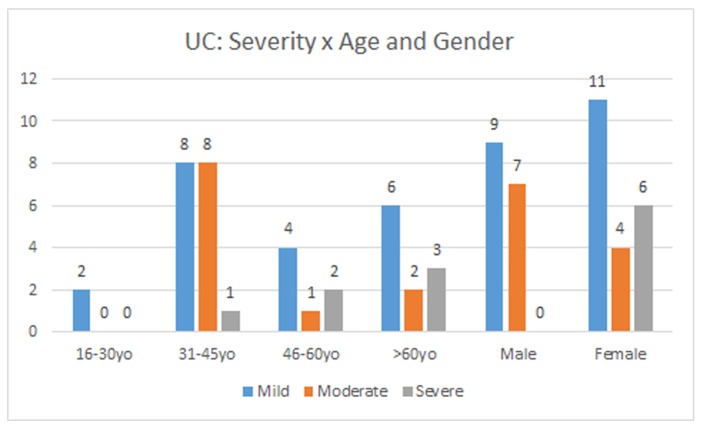
UC severity according to gender and age.

The mild and moderate forms of the UC had its highest incidence in the age group of 31 - 45 years old and the severe form in the over 60 years olds. The mild (55%) and severe (100%) forms of the disease affected women more frequently, while the moderate form (64%) affected mostly men (Fig. 2).

## Discussion

IBDs are closely related to one another; however, epidemiological studies show clinical distinctions [23, 24]. In this study, the proportion of patients with UC to those with CD was 1.7:1, as observed in other studies [4, 6, 7]. In hospitalized patients, this proportion has changed, being justified by the fact that CD has a more severe course favoring a higher need of hospitalization and interventions [22, 23].

We observed significance (P < 0.05) when analyzing the age, place of residence and educational background in the studied group. The age group of 31 - 45 years old has been more prevalent and only four patients were under 16 years old. Approximately 20% of the patients with IBD developed the symptoms as a child or adolescent [1, 25-27]. We also observed through this study that the CD is associated with younger people (under 25) and the UC with older patients (over 25 years). Studies suggest a bimodal age distribution for both diseases with a possible second peak between 50 and 80 years old [28]. The male/female distribution was 1:1.86 to CD and 1:1.35 for UC. The UC affects 60% of men, and CD affects 20-30% women, especially in high incidence areas [1, 29].

Few studies [21] describe prevalence associated with urban or rural origins of the patient. Urban origin (P < 0.03) and the educational background (P < 0.01) of these patients were statistically significant. It is possible that this fact favors the search for a faster diagnosis. Patients reported skin color as brown. There was no significant difference between CD and UC. Black and Hispanic populations have low incidence of IBD. Environmental factors, lifestyle and genetic differences may correlate to these factors [30, 31]. There was no significant difference between patients with CD and UC as for the family income and employment situation.

At the time of data collection, over 50% of the patients were former smokers. There was some predominance of smoking habit in patients with CD. We could not identify relevant data regarding the number of cigarettes/day being linked to the onset of the disease, which made it impossible to correlate smoking with the location and extent of intestinal involvement and the complicated forms of CD and UC. We observed significant deterioration in individuals with CD during clinical follow-up of patients with a smoking history, whereas those may have a worse outcome compared to those who never smoked.

Tobacco addiction is a known factor in the incidence of CD [26]. Smokers are more likely to develop CD than those who never smoked. We discussed the protective effect of smoking in the course of UC [31, 32].

In this study, people under 25 years old were most affected with CD compared to those who had UC (> 25 years old). The patient’s age when symptoms started was statistically significant (P < 0.01). The time interval between onset of symptoms and the diagnosis was between 5 and 10 years and it was lower in patients with UC compared to those with CD. Although CD has slower evolution, we wonder whether that is due to the complications that can manifest even before the definitive diagnosis, either by misdiagnosis or as part of the natural course of the disease. Studies [32] state that confirmation of diagnosis occurs around 10 years after the diseases’ onset.

The course of IBD differs between its presentation forms. Patients may experience symptoms for years before diagnosis [33]. In UC symptoms gradually manifest and progressively become more severe, which may lead to acute episodes of rectal bleeding lasting weeks or months [6]. Unlike UC, CD evolves in the first 10 years without complications. UC can evolve in the first 10 years with low surgical intervention and disease remission. However, colonic involvement within 5 - 10 years [32] can occur in about 20% of patients.

There was more family history of IBD among patients with CD, but it was not significant statistically. Studies [6, 7] indicate a higher frequency in the UC, while other authors [33] suggest that both forms of the disease have a genetic background.

Appendectomy was statistically significant (P < 0.02) related to patients with CD. According to studies [7, 26, 34, 35] appendectomy may exert a protective effect on lower incidence of UC. In our study appendectomy was only associated with CD.

In the population sample, the inflammatory involvement B1 was predominant in CD. The data revealed that 30% of patients were affected by abscess and fistula. It is possible that up to 45% of patients develop a leak before the diagnosis of CD [7, 36]. The disease behavior change in the natural history of CD consists in the inflammatory disease (B1) evolving to stenotic (B2) or penetrating (B3) manifestations [17].

As for the location, ileocolic CD (L1) was the most frequent, being similar to other studies [36]. Approximately 80% of the patients have involvement of the small intestines, and a third is exclusively ileitis. The ileocolic region (50%) and colon (20%) account for the other locations of the disease. As the extent of the UC, proctitis (E1) was predominant. Studies [36, 37] indicate that proctitis has a 50% chance of compromising its length and the proximal disease that affects the sigmoid colon has 9% chance of progressing to pancolitis.

The presence of a palpable mass was significant (P < 0.01) in individuals with CD. In patients with UC exclusive rectal bleeding was statistically significant (P < 0.04). The abdominal pain and tenderness, tenesmus, liquid diarrhea, bloody diarrhea and mucus in the stool were constant features in this group.

Osteoarthicular EIMs were found both in CD patients and in those with UC. Arthritis was more prevalent, confirming literature data [18-21]. Fifteen patients had at least one osteoarthicular manifestation. EIMs were more frequent in CD which confirms how much you should be aware of these patients' signs and symptoms. Clinical manifestations in the upper gastrointestinal tract [38], dermatological and vascular were observed only in patients with CD. Low percentage of autoimmune hepatic manifestations was observed in the sample, similar to what is seen in literature [39, 40].

Conjunctivitis was significant statistically (P < 0.04) in patients with CD. Uveitis and episcleritis are frequent ocular manifestations of IBD, although the scleritis, iritis and conjunctivitis may also be associated [41].

Systemic manifestations were present in both forms of IBD. The severity of symptoms can vary according to frequency of defecation, presence of bleeding, fever, fatigue, abdominal pain and persistent weight loss. Our data showed that the mild and severe form of the disease affected mostly women over 25 years old, while men expressed moderate symptoms.

### Conclusions

Data revealed that IBD is most prevalent in women. Age, origin and level of education may influence early diagnosis. CD was associated with younger individuals, females, urban origin, family history, time interval between symptoms and diagnosis, smoking habits, appendectomy, and surgical complications, palpable mass and greater number of EIMs. In UC prevailed women, the time interval between symptom and diagnosis was late and severe forms of the disease occurred at older ages, setting the gradual and progressive course of the disease. Systemic manifestations were present in both forms of IBD. Demographic and clinical aspects were similar to the literature, although data differed as to the time interval between the onset of symptoms and diagnosis, smoking habits, appendectomy and severity of UC for age and gender. New research should better elucidate this information.
